# Impact of total marrow/lymphoid irradiation dose to the intestine on graft-versus-host disease in allogeneic hematopoietic stem cell transplantation for hematologic malignancies

**DOI:** 10.3389/fonc.2022.1035375

**Published:** 2022-12-08

**Authors:** Simonetta Saldi, Christian Paolo Luca Fulcheri, Claudio Zucchetti, Amr Mohamed Hamed Abdelhamid, Alessandra Carotti, Antonio Pierini, Loredana Ruggeri, Sara Tricarico, Marino Chiodi, Gianluca Ingrosso, Vittorio Bini, Andrea Velardi, Massimo Fabrizio Martelli, Susanta Kumar Hui, Cynthia Aristei

**Affiliations:** ^1^ Section of Radiation Oncology, Hospital of Santa Maria della Misericordia, Perugia, Italy; ^2^ Medical Physics, Hospital of Santa Maria della Misericordia, Perugia, Italy; ^3^ Radiation Oncology Section, Department of Medicine and Surgery, University of Perugia and Perugia General Hospital, Perugia, Italy; ^4^ Department of Oncology and Nuclear Medicine, Faculty of Medicine, Ain Shams University, Cairo, Egypt; ^5^ Division of Hematology and Clinical Immunology, Department of Medicine, University of Perugia, Perugia, Italy; ^6^ Radiology Unit, S. Maria Della Misericordia Hospital, Perugia, Italy; ^7^ Internal Medicine, Endocrine and Metabolic Science Section, University of Perugia, Perugia, Italy; ^8^ Department of Radiation Oncology, City of Hope National Medical Center, CA, United States

**Keywords:** TMLI, graft-versus-host disease, tomotherapy, intestine dose radiotherapy, HSCT = hematopoietic stem cell transplant, intestinal acute graft-versus-host disease

## Abstract

**Background and purpose:**

Graft-versus-host disease (GvHD) is a leading cause of non-relapse mortality in patients undergoing allogeneic hematopoietic stem cell transplantation. The Perugia Bone Marrow Transplantation Unit designed a new conditioning regimen with total marrow/lymphoid irradiation (TMLI) and adaptive immunotherapy. The present study investigated the impact of radiotherapy (RT) doses on the intestine on the incidence of acute GvHD (aGvHD) in transplant recipients, analyzing the main dosimetric parameters.

**Materials and methods:**

Between August 2015 and April 2021, 50 patients with hematologic malignancies were enrolled. All patients underwent conditioning with TMLI. Dosimetric parameters (for the whole intestine and its segments) were assessed as risk factors for aGvHD. The RT dose that was received by each intestinal area with aGvHD was extrapolated from the treatment plan for each patient. Doses were compared with those of the whole intestine minus the affected area.

**Results:**

Eighteen patients (36%) developed grade ≥2 aGvHD (G2 in 5, G3 in 11, and G4 in 2). Median time to onset was 41 days (range 23–69 days). The skin was involved in 11 patients, the intestine in 16, and the liver in 5. In all 50 TMLI patients, the mean dose to the whole intestine was 7.1 Gy (range 5.07–10.92 Gy). No patient developed chronic GvHD (cGvHD). No dosimetric variable emerged as a significant risk factor for aGvHD. No dosimetric parameter of the intestinal areas with aGvHD was associated with the disease.

**Conclusion:**

In our clinical setting and data sample, we have found no clear evidence that current TMLI dosages to the intestine were linked to the development of aGvHD. However, due to some study limitations, this investigation should be considered as a preliminary assessment. Findings need to be confirmed in a larger cohort and in preclinical models.

## Introduction

Hematopoietic stem cell transplantation (HSCT), the most effective post-remission treatment for acute leukemia (AL), is indicated for patients in ≥ second complete remission (CR) or in first CR with unfavorable cytogenetics and molecular markers (intermediate–high-risk AL) (1). HSCT achieves its effect through the conditioning regimen’s myeloablation and the graft’s elimination of residual leukemic cells [graft *vs*. leukemia (GvL) effect]—thanks to its donor T-lymphocyte content (2). On the other hand, this can cause graft-versus-host disease (GvHD), a leading cause of non-relapse mortality (3).

GvHD is usually distinguished as acute or chronic, each with a different underlying mechanism. Acute GvHD (aGvHD) includes a combination of symptoms and signs that usually occur in the first 100 days posttransplant but may have a later onset. Chronic GvHD (cGvHD), the most frequent cause of late non-relapse morbidity and mortality, might affect several organs, determining functional impairment (4, 5). Approximately 30%–50% of HSCT recipients develop aGvHD typically affecting the skin, gastrointestinal (GI) tract, and liver, and 10%–70% are affected by cGvHD (6, 7) that manifests like autoimmune diseases such as eosinophilic fasciitis or scleroderma-like skin disease (8).

Risk factors for aGvHD include unrelated or alternative donors, donor parity, donor–recipient sex mismatch, elderly recipient, advanced-stage disease, low regulatory T-cell content in the graft (9). Furthermore, conditioning regimens that included total body irradiation (TBI) were associated with a higher incidence of aGvHD than chemotherapy alone (10–12). On the other hand, the large Forum Randomized Controlled Trial did not show significant differences in aGvHD in children after chemotherapy or 6 × 2 Gy TBI conditioning (13). The robust GI structure and function in young patients might have enabled them to tolerate higher GI doses with TBI.

Radiation dose correlated with aGvHD severity. In a mouse model, Hill et al. (14) showed that a higher TBI dosage (13 Gy *vs*. 9 Gy) led to greater intestinal damage and more severe GvHD. High-dose TBI (15.75 Gy *vs*. 12 Gy) was also associated with more aGvHD in a clinical study by Clift et al. (15). In a large series of patients who had received matched or mismatched stem cell transplantation, TBI >12 Gy emerged as a risk factor for GvHD (44% with doses >12 Gy *vs*. 28% with 0–12 Gy, p = 0.001) (16).

Radiation-related damage to the GI tract plays a major role in aGvHD development and its systemic involvement by triggering and propagating the cytokine storm (17). Critically, the TBI dose can injure the intestinal mucosa, inducing inflammation and promoting translocation of inflammatory stimuli, thus further damaging the GI tract. Furthermore, the conditioning regimen injures tissues and activates inflammatory cytokines tumor necrosis factor (TNF)-α and interleukin (IL)-1. Tissue damage is amplified by donor T-cell activation leading to IL-2 and interferon (IFN)-γ secretion. Intestinal mucosal damage increases the release of lipopolysaccharides and stimulates cytokine production by lymphocytes and macrophages in the GI tract and by keratinocytes, dermal fibroblasts, and macrophages in the skin (18).

In order to reduce the radiotherapy (RT) dose to the GI tract and to other organs at risk (OARs) of toxicity, such as the lungs, heart, and kidneys (19), total marrow irradiation (TMI) and total marrow/lymphoid irradiation (TMLI) were introduced into the conditioning regimens (20–23). Unlike TBI, the radiation target volumes for TMI is only the skeleton, while total lymphoid irradiation (TLI) is targeted at major lymph node chains and non-lymphoid organs, such as the spleen and liver. Preclinical data in a murine model confirmed that, compared with TBI, TMI reduced the dose to the GI tract and thus the risk of aGvHD-mediated tissue damage (24). In a retrospective cohort analysis, Haraldsson et al. (25) reported less aGvHD after TMI with tomotherapy than two-dimensional (2D) TBI.

The present study assessed whether clinical parameters and the dose delivered to the intestine were risk factors for aGvHD in patients undergoing TMLI in the conditioning regimen for HSCT.

## Patients and methods

Between August 2015 and April 2021, this prospective observational study recruited 50 patients [median age 56 years, range 23–70 years; 33 men; 17 women; 44 with acute myeloid leukemia (AML), 3 with acute lymphoid leukemia (ALL), and 3 with myelodysplastic syndrome]. The study was conducted in accordance with the 1975 Helsinki Declaration, as revised in 2000, and all patients provided written informed consent. The indication for TMLI was age >50 years old. Six unfit patients (i.e., with comorbidities that precluded TBI) who were <50 years old also received TMLI. Before conditioning, no patient was affected by GI disturbances. **Table 1** reports details of these 50 patients.

**Table 1:** Details of 50 patients who underwent a TLMI-based conditioning regimen to hematopoietic stem cell transplantation (HSCT).

**Sex**	
Male	33
Female	17
**Age (years)**	
Median	56
Range	23–70
**Hematologic malignancies**	
Acute myeloid leukemia	44
Acute lymphoid leukemia	3
Myelodysplastic syndrome	3
**Genetic risk stratification at diagnosis**	
Favorable	4
Intermediate	21
Adverse	22
Missing information	3
**Disease status at HSCT**	
First complete remission (CR)	24
≥ Second CR	21
Advanced	5
**Minimal residual disease (MRD)**	
MRD positive	33
No MRD	17

### Radiotherapy

TMLI was administered to all patients by helical tomotherapy in nine fractions delivered twice daily for 4.5 consecutive days. Using a Simultaneous Integrated Boost (SIB) procedure, target volumes were skeletal bones for TMI (total dose 13.5 Gy) and major lymph node chains and spleen for TLI (total dose 11.5 Gy). Patients with ALL received 13.5 Gy to the brain. All patients were reproducibly immobilized using a vacuum cushion and a 5-point open-face thermoplastic mask for the head, neck, and shoulders. Since the tomotherapy unit treats up to 135 cm in length, treatment was split into two plans: the upper, comprising approximately from the vertex to the knees, and the lower, from approximately the toes to the hip bone. All patients underwent two computed tomography (CT) scans, using 10-mm slice thickness, in opposite directions, with the patient rotated through 180 degrees. To reach an acceptable dose homogeneity in the junction region of the plans, a controlled dose gradient was created using five regions inside the overlap volume.

On the CT images, one expert radiation oncologist (SS) contoured OARs (**Table 1**) and target structures using the Pinnacle TPS v.16 (Philips) contouring tool. Before transferring images and RT structures to the Accuray^®^ Planning Station 5.1.1 for plan optimization, expert medical physicists (CZ, CF) reviewed the volumes and created planning regions of interest (ROIs) (e.g., remaining volume at risk, healthy lungs, junctions) using a fully automated Pinnacle script.

Plan setup and optimization were done using a dedicated protocol with the following parameters: “fine” dose calculation grid, 5.02-cm field width; fixed jaw mode. For the upper and lower plans, planning modulation factors were in the range of 3.0–3.7 and 2.0–2.5, respectively; the pitch was in the range of 0.32–0.43 for the upper plan and was set at 0.287 for the lower. The pitch value of the upper plan was selected to minimize the thread effect, especially for off-axis targets such as the arms, and to reach a compromise between dose homogeneity and gantry period.

Treatment planning system planning optimization goals were set so that 100% of the prescribed dose covered 60% of the planning target volume (PTV) and at least 95% of the prescribed dose covered 90% of the PTV.

The optimization procedure focused on dose reduction to the main OARs, i.e., the heart, bowel, liver, lungs, and kidneys, and was iterated at least 500 times. Treatment plans were assessed and approved according to the following criteria: individual patient factors and needs, dose distribution conformity and homogeneity (as visually assessed), hot spots within the target, adequate treatment time for patient compliance, achievement of target coverage objectives, and average doses to the OARs in accordance with our center’s reference values (**Table 2**). Treatment plans were satisfactory when doses fell within the ranges shown in **Table 2**. By respecting these constraints, no plan has to date fallen outside of our median range. If it were to happen, the plan would be redone.

**Table 2:** Organs at risk (OARs) average and range of median doses.

Organ	Median dose in Gy Average (Range)*
Anus	7.2 (3.14 – 14.81)
Bladder	8.98 (6.44 – 13.37)
Brain	8.87 (7.74 – 13.57)
Esophagus	11.4 (8.47 – 13.9)
Heart	6.35 (5.30 – 9.04)
Kidney (left)	6.27 (5.08 – 9.3)
Kidney (right)	5.69 (4.42 – 8.85)
Large bowel	7.93 (6.34 – 11.73)
Lens (left)	3.2 (1.85 – 5.19)
Lens (right)	3.3 (1.92 – 4.91)
Liver	7.72 (5.7– 10.21)
Lung (left)	8.97 (6.35 – 10.98)
Lung (right)	8.7 (6.31 – 10.65)
Oral cavity	8.83 (5.59 – 12.45)
Rectum	7.75 (5.55 – 11.66)
Small bowel	6.43 (5.61 – 10.59)
Stomach	8.39 (5.5 – 12.81)
Thyroid	10.95 (7.31 – 13.94)

*Average and range (minimum and maximum median dose) of median doses in 75 patients with diverse hematologic malignancies. Values derive from a retrospective analysis of our entire series of 75 TMLI patients, starting in 2015. They were gradually reduced over time to account for updates in skills and for values reported elsewhere.

### Transplantation procedure

**Figure 1:**
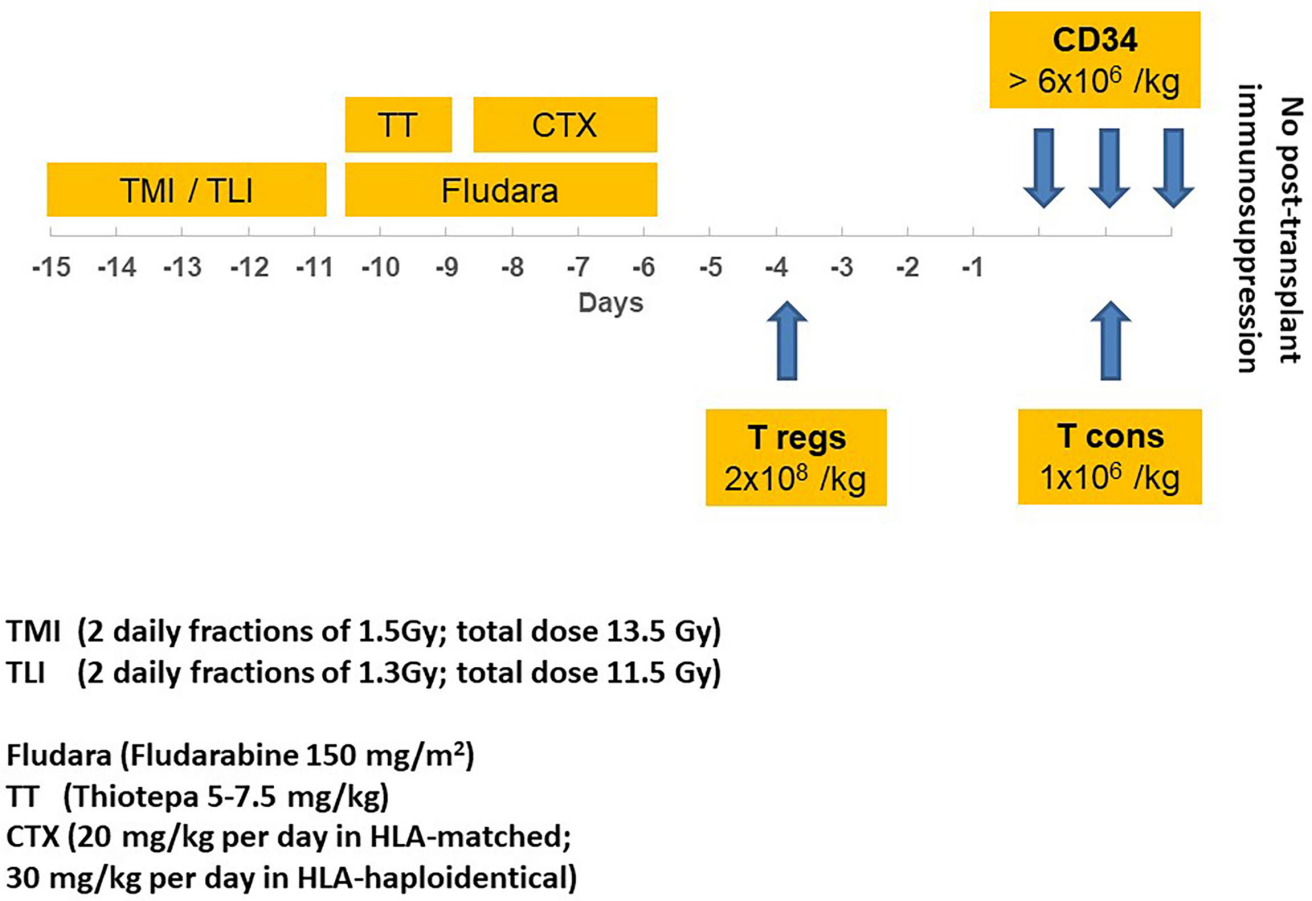
Transplantation schema, illustrating TMI/TLI* irradiation, drugs, timing, and immunotherapy before hematopoietic stem cell transplantation with CD34^+^ cells. *A SIB procedure was used to deliver different TMI and TLI total doses (respectively, 13.5 Gy and 11.5 Gy); TMI, total marrow irradiation; TLI, total lymphoid irradiation; CTX, cyclophosphamide; Fludara, fludarabine; T regs, regulatory T cells; T cons, conventional T cells.

**Figure 1** illustrates the conditioning regimen and graft composition, showing TMLI was followed by thiotepa (5–7.5 mg/kg), fludarabine (150 mg/m^2^), and cyclophosphamide (20 mg/kg per day in Human leukocyte antigens (HLA)-matched HSCT; 30 mg/kg per day in HLA-haploidentical HSCT). Donors were HLA-matched related for 11 patients and HLA-haploidentical mismatched family members for 39. Apheresis procedures were described in full elsewhere (23). All patients received an infusion of 2 × 10^6^/kg donor regulatory T cells (Tregs) on day -4 followed by 1 × 10^6^/kg conventional T cells (Tcons) on day -1. A megadose of >6 × 10^6^/kg positively selected CD34^+^ hematopoietic progenitor cells was infused on day 0.

### Prophylaxis for Acute graft-versus-host disease (aGvHD) and infections

aGvHD prophylaxis consisted of *ex vivo* T-lymphocyte depletion by positive immunoselection of CD34^+^ peripheral hematopoietic progenitor cells and donor Tregs. No pharmaceutical immunosuppressive therapy was given posttransplant. All patients received antibacterial, antifungal, antiviral, and anti-*Pneumocystis carinii* prophylaxis. GI status and all symptoms were registered in each patient’s chart before, during, and after TMLI and after transplantation.

### aGvHD assessment

Engrafted patients who survived more than 30 days were evaluable for aGvHD, which was assessed according to the Glucksberg score (26). The grade was determined by the worst disease stage in any organ. Diagnosis of intestinal aGvHD was confirmed by means of a CT scan in the acute phase of intestinal inflammation. aGvHD areas were defined by indicative radiological signs (i.e., luminal dilation with small bowel wall thickening (“ribbon sign”) and air/fluid levels suggesting an ileus) (27).

### Dosimetric analysis

**Figure 2:**
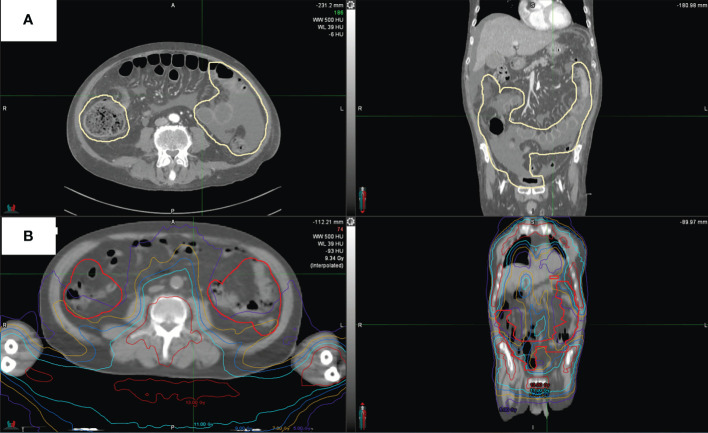
CT scan of one representative patient. **(A)** shows intestinal areas with aGvHD as outlined in pale yellow. **(B)** shows aGvHD as contoured on this patient’s original treatment plan and dose distribution.

For the whole intestine and separately for the small intestine, large intestine, duodenum, sigmoid, and rectum, the following dosimetric parameters were analyzed: dose received by 5 cc (D5cc), 10 cc (D10cc), 30 cc (D30cc), 50 cc (D50cc), 80 cc (D80cc); mean dose (Dmean); maximum dose (Dmax); volumes that received 5 Gy (V5Gy), 7 Gy (V7Gy), 9 Gy (V9Gy), 11 Gy (V11Gy), 13 Gy (V13Gy), or more. For the purposes of this study, an expert radiologist (MC) contoured on the planning CT images the areas that had radiological signs indicative of aGvHD on diagnostic CT scans, an example is provided in **Figure 2**. The RT dose that was received by each intestinal area with aGvHD (V5Gy, V7Gy, V9Gy, V11Gy, V13Gy, Dmin, Dmean, Dmax) was extrapolated from the treatment plan for each patient. Doses to each intestinal area with aGvHD were compared with those of the whole intestine minus the affected area.

### Statistical analyses

Dosimetric parameters as above and clinical variables {age, body mass index (BMI), residual disease at transplant [minimal residual disease (MRD) >0.1% blasts at cytofluorimetric analysis of bone marrow]} were assessed as risk factors for aGvHD. The Shapiro–Wilk test checked if variables were normally distributed. As they were non-normally distributed, data were expressed as median (min–max). The Mann–Whitney and Wilcoxon tests were used for continuous/discrete variables, i.e., independent and paired data, respectively. The chi-square test with Yates’ correction or Fisher’s exact test was used to compare categorical variables. Univariate estimates of time-related outcome measures for survival curves were determined using the Kaplan–Meier product-limit method. All statistical analyses were performed using IBM-SPSS^®^ version 26.0 (IBM Corp., Armonk, NY, USA, 2019). In all analyses, a two-sided p-value ≤0.05 was considered significant.

## Results

All 50 patients achieved primary sustained engraftment with full donor-type chimerism. **Table 3** summarizes outcomes.

**Table 3:** Outcomes of TMLI and hematopoietic stem cell transplantation in 50 patients with hematologic malignancies.

Engraftment	50 patients
**Infections in 30 patients**	
Pulmonary	26
Gastrointestinal	21
Genitourinary	9
**Acute GvHD in 18 patients**	
Intestinal	16
Liver	5
Skin	11
**Chronic GvHD**	0
**Follow-up in months**	34 (1–80)
**Transplant-related mortality (TRM)**	16% (median time to death: 5.2 months)
Leukemia-free survival*	74.4%

* at 60 months.

In the first 3 months posttransplant 30/50 (60%) transplant recipients developed an infectious complication that was defined as organ damage coupled with fever (26 pulmonary, 21 GI, 9 genitourinary). Infections were due to bacteria, fungi, and viruses, but infectious agents were not always identified.

All patients were evaluable for aGvHD. Eighteen patients (36%) developed grade ≥2 aGvHD (G2 in 5, G3 in 11, and G4 in 2). Infections and other sources of inflammation were ruled out as underlying causes. The median time to onset was 41 days (range 23–69 days). The skin was affected in 11 patients and the liver in 5. At diagnosis of aGvHD, the CT scan was positive for intestinal involvement in 16/18 patients. All 18 patients received steroids as first-line therapy. Second-line treatment was administered to 13 patients either for early steroid withdrawal or to achieve a better aGvHD response. Agents included cyclosporine, anti-thymocyte globulin, extracorporeal photopheresis, and ruxolitinib. aGvHD resolved in 16/18 patients who fully withdrew from immunosuppressive treatments. At a median follow-up of 34 months (range 1–80 months), no patient has developed cGvHD.

In the 18 patients with aGvHD, neither age (p = 0.666), BMI (p = 0.495), nor residual disease (p = 0.653) at transplant was found to be a risk factor.

In the entire cohort of 50 patients, the mean TMLI dose to the whole intestine was 7.1 Gy (range 5.07–10.92 Gy). No dosimetric variable emerged as a significant risk factor for aGvHD (**Table 4**). No dosimetric parameter of the intestinal areas with aGvHD was associated with the disease (**Table 5**).

**Table 4:** Impact of dose delivered to the whole intestine and its segments on aGvHD.

Dosimetric variables	aGvHD (yes) (n = 18)		aGvHD (not) (n = 32)		
	Median	Min–max	Median	Min–max	p-value
D5cc duodenum	11.2	8.43 - 13.00	11.2	7.90 - 12.97	0.5783
D10cc duodenum	10.5	7.75 - 12.54	10.7	7.39 - 12.23	0.7387
D30cc duodenum	8.3	4.56 - 11.73	8.6	5.15 - 10.59	0.751
V5Gy duodenum	57.9	24.37 - 640.71	51.4	17.85 - 95.20	0.1693
V7Gy duodenum	39.3	14.37 - 443.80	39.3	15.53 - 85.02	0.3631
V9Gy duodenum	23.7	2.00 - 293.10	23.6	0.22 - 61.44	0.5992
V11Gy duodenum	5.8	0.00 - 125.17	6.8	0.00 - 23.32	0.7311
V13Gy duodenum	0.0	0.00 - 4.98	0.0	0.00 - 4.87	0.9145
D5cc large intestine	13.2	11.78 - 14.11	13.1	11.44 - 14.28	0.233
D10cc large intestine	13.0	11.64 - 13.94	13.0	11.32 - 14.05	0.3575
D30cc large intestine	12.7	11.28 - 13.55	12.5	10.99 - 13.73	0.3903
D50cc large intestine	12.4	10.89 - 13.29	12.1	10.69 - 13.62	0.284
D80cc large intestine	12.0	10.20 - 13.06	11.7	10.26 - 13.51	0.2841
V5Gy large intestine	1,498.3	504.76 – 2,689.28	1,161.4	538.29 – 2,317.66	0.531
V7Gy large intestine	931.0	323.83 – 1,856.46	690.5	352.30 – 1,606.63	0.2331
V9Gy large intestine	540.0	180.38 – 1,234.07	379.6	176.36 – 1,369.81	0.2331
V11Gy large intestine	240.1	46.12 - 761.86	156.2	29.63 - 836.66	0.2751
V13Gy large intestine	11.6	0.00 - 92.88	8.9	0.00 - 217.02	0.2369
D5cc rectum	10.7	5.83 - 13.01	10.4	4.19 - 12.18	0.413
D10cc rectum	9.3	4.92 - 12.50	8.5	3.81 - 11.62	0.3122
V5Gy rectum	45.0	9.05 - 135.08	38.4	2.14 - 162.84	0.4669
V7Gy rectum	18.9	0.83 - 82.28	18.4	0.28 - 57.04	0.6712
V9Gy rectum	10.8	0.00 - 77.11	8.4	0.00 - 43.59	0.3902
V11Gy rectum	4.1	0.00 - 76.63	3.3	0.00 - 19.72	0.2027
V13Gy rectum	0.1	0.00 - 5.12	0.0	0.00 - 1.07	0.0769
D5cc sigmoid	11.8	10.26 - 13.37	12.0	9.33 - 13.33	0.3026
D10cc sigmoid	11.4	7.47 - 13.01	11.6	8.09 - 13.01	0.1725
D30cc sigmoid	9.9	4.37 - 12.13	10.0	6.01 - 11.90	0.908
V5Gy sigmoid	73.7	11.23 - 159.12	65.7	31.00 - 240.90	0.7927
V7Gy sigmoid	52.6	10.47 - 139.73	54.8	18.30 - 147.52	0.8241
V9Gy sigmoid	35.7	7.11 - 102.37	37.2	6.09 - 84.98	0.4793
V11Gy sigmoid	16.3	0.22 - 58.16	17.7	0.37 - 43.34	0.4669
V13Gy sigmoid	0.5	0.00 - 10.26	0.4	0.00 - 10.12	0.7799
D5cc small intestine	12.3	11.55 - 13.75	12.2	11.09 - 13.75	0.7694
D10cc small intestine	12.0	11.40 - 13.55	11.8	10.83 - 13.70	0.5991
D30cc small intestine	11.6	10.77 - 13.11	11.4	10.18 - 13.60	0.6934
D50cc small intestine	11.2	10.36 - 12.78	11.2	9.84 - 13.55	0.6565
D80cc small intestine	10.8	9.92 - 12.42	10.8	9.27 - 13.43	0.642
V5Gy small intestine	1,167.3	595.25 – 3,061.58	1,071.2	522.12 – 3,068.37	0.5715
V7Gy small intestine	589.4	271.24 – 1,069.72	556.7	255.00 – 1,166.77	0.8241
V9Gy small intestine	250.3	143.99 - 574.82	258.0	95.19 - 877.30	0.9035
V11Gy small intestine	68.1	19.69 - 266.15	63.6	6.46 - 572.45	0.6134
V13Gy small intestine	0.3	0.00 - 35.95	0.7	0.00 - 165.66	0.5763
D5cc whole intestine	13.3	11.85 - 14.11	13.2	11.68 - 14.28	0.2932
D10cc whole intestine	13.1	11.74 - 13.96	13.0	11.58 - 14.06	0.3472
D30cc whole intestine	12.8	11.54 - 13.57	12.6	11.30 - 13.76	0.363
D50cc whole intestine	12.5	11.37 - 13.32	12.3	11.08 - 13.69	0.3848
D80cc whole intestine	12.1	11.14 - 13.09	12.0	10.78 - 13.62	0.3319
V5Gy whole intestine	2,802.6	1,155.91 – 5,925.41	2,380.1	1,102.50 – 5,011.60	0.492
V7Gy whole intestine	1,537.8	636.36 – 3,042.76	1,286.3	647.01 – 2,653.46	0.1822
V9Gy whole intestine	834.8	380.51 – 1,636.88	709.8	313.04 – 2,202.96	0.2577
V11Gy whole intestine	314.7	99.61 - 851.36	247.6	57.91 – 1,430.08	0.1513
V13Gy whole intestine	12.7	0.00 - 99.62	11.6	0.00 - 387.55	0.3472

Statistical analyses were based on Mann–Whitney test; significance was set at p < 0.05.

**Table 5:** RT dose to intestinal areas developing aGvHD vs. RT dose to non-affected intestine.

Dosimetric variables	GvHD		Gut without GvHD		
	Median	Min–max	Median	Min–max	p-value
V5Gy	722.8	186.99 – 3,206.06	1,703	391.05 – 3,829.56	0.3635
V7Gy	469.680	93.32 – 2,124.64	848.1	218.46 – 1,865.70	0.3635
V9Gy	219.380	39.68 – 1,196.45	628.5	146.48 – 1,075.89	0.3066
V11Gy	111.81	2.76 - 543.21	253.3	40.85 - 489.29	0.2115
V13Gy	8.340	0.00 - 52.46	8.83	0.00 - 68.16	0.2115
Dmin	3.100	2.13 - 6.33	3.09	2.00 - 4.18	0.1318
Dmean	7.280	5.84 - 10.47	7.52	5.06 - 8.86	0.2012
Dmax	13.800	12.22 - 15.32	13.98	12.62 - 14.78	0.256

Statistical analyses were based on Wilcoxon signed rank test; significance was set at p < 0.05.

When 13 patients with G3 and G4 aGvHD were compared with 32 patients who were aGVHD-free, no dosimetric parameter emerged as significant.

## Discussion

TMLI was recently introduced into the Perugia Unit’s conditioning regimen as an alternative to TBI in association with a graft containing Tregs and Tcons (28). The inoculum content of a megadose of T cell-depleted CD34^+^ cells and donor Tregs constituted the only prophylaxis for aGvHD that otherwise would have been triggered by the Tcon content. Indeed, evidence from murine haploidentical transplant models showed that confusion of Tregs with Tcons prevented lethal aGvHD by suppressing alloreactive T-cell proliferation in the lymph nodes and non-lymphoid tissues, i.e., the skin, liver, gut, and lung. Since the expansion of non-alloreactive T cells was not inhibited, immunological reconstitution proceeded unhindered (29–32).

The choice of TMLI or TBI was dictated by the patient’s age and condition. The present study protocol was designed to offer HSCT to patients with hematologic malignancies who were ineligible for TBI, i.e., 44 patients because of age, which was >50 years, and 6 younger patients who had comorbidities that were counterindications to TBI. The study’s inclusion criteria precluded a TBI control group.

In a previous series of high-risk AML patients, RT tailoring in the conditioning regimens to suit individual needs was associated with an exceptional 75% of cGvHD/relapse-free survival, despite T-cell depletion strategies. Grade ≥2 aGvHD occurred in 15 patients, i.e., in 3/19 (16%) who underwent TBI and surprisingly in 12/31 (39%) who underwent TMLI (33). Although older age was hypothesized to have been a factor in the development of aGvHD (33), it did not emerge as a risk factor in the present series, with ages ranging from 23 to 70 years old. The discrepancy may be resolved in the future by conducting a prospective study with the same eligibility criteria.

The present study focused on radiation-related damage to the intestine, as it was reported to play a major role in the development of aGvHD and its systemic involvement by propagating the cytokine storm (17). We found no evidence that TMLI dosages to the intestine were linked to the development of aGvHD in HSCT recipients for the current level of dose exposure (mean dose 7.1 Gy; range: 5.07–10.92 Gy). Risk factors other than RT may have triggered aGvHD in our patients. One culprit may have been the Tcon content in the graft as T cell-depleted grafts were associated with a lower aGvHD incidence (9%) in our previous series of HSCT patients who were conditioned with single-dose or hyperfractionated TBI (34).

We further investigated whether RT doses to the entire intestine or its diverse segments were risk factors for aGvHD, taking into account CT evidence of aGvHD and radiosensitivity variations in the different segments. In fact, some intestinal cells, like potential stem cells, are highly radioresistant and are activated at high-dose (9 Gy) irradiation. Like these potential stem cells, cells contributing to the recovery of crypts and highly apoptosis-sensitive cells are also found in different percentages in the large and small intestine, providing different radiosensitivity indices and making the small intestine more radiosensitive than the colon and rectum (35).

The present results did not identify any dosimetric variable that correlated with aGvHD in the whole intestine or its segments. Furthermore, no parameter emerged as linked to aGvHD even when the analysis was restricted to the radiological area where damage was visible on the CT scan.

Several hypotheses were explored to account for the present results. In the first instance, RT dose to the GI, tissue damage, and aGvHD occurrence are possibly nonlinear. Our clinical priority of administering low RT doses to the intestine so as to preserve intestinal function and prevent aGvHD may have been transformed into a drawback, as low doses were administered to the whole intestine and its segments, thus making a significant finding hard to emerge. On the other hand, we have to admit that the large dose variations in the intestine and the small cohort of patients further limit resolving the difference. Secondly, it was difficult to define the exact RT dose for the whole intestine and its segments. Indeed, organ motion and natural variations in volume impact upon dose delivery and intertreatment and intratreatment sessions. Intriguingly, several preclinical studies showed that commensal bacteria influenced the pathophysiology of GvHD (36). When evaluating long-term changes in gut microbiota after TBI in a murine model, Zhao et al. (37) demonstrated quantitative and qualitative changes in microbial diversity. The results of ongoing trials of targeted modulation strategies in HSCT recipients (36, 38) are eagerly awaited.

Finally, contributing to the complexity of untangling the role of RT is a combination of immune modulation as induced by GI radiation and adoptive therapy with Tcons and Tregs. Thus, a preclinical model will be a helpful guide in understanding the role of TMI in aGvHD. Indeed, we have already observed that lowering the radiation dose (~4 Gy) to the GI attenuated tissue damage, with less donor T-cell traffic to the GI system that resulted in reduced aGvHD.

Ultimately, since the present small sample size of 50 patients with 18 cases of aGvHD may account for our lack of significance, this investigation should be considered as a preliminary assessment. Recruitment is continuing, as are studies in preclinical models, in an attempt to explore other potential triggers of aGvHD and provide more definitive findings about its prevention.

## Data availability statement

The raw data supporting the conclusions of this article will be made available by the authors, without undue reservation.

## Ethics statement

Ethical review and approval was not required for the study on human participants in accordance with the local legislation and institutional requirements. The patients/participants provided their written informed consent to participate in this study.

## Author contributions

SS, CF, CZ, AA, AP, MC, GI, SH and CA contributed to conception and design of the study. SS, CF and CZ organized the database. VB performed the statistical analysis. SS, CA and SH wrote the first draft of the manuscript. CF, AP, CZ, AA wrote sections of the manuscript. All authors contributed to manuscript revision, read, and approved the submitted version.
